# Communicative predictions can overrule linguistic priors

**DOI:** 10.1038/s41598-017-17907-9

**Published:** 2017-12-14

**Authors:** Leon O. H. Kroczek, Thomas C. Gunter

**Affiliations:** Max Planck Institute for Human Cognitive and Brain Sciences, Leipzig, Germany

## Abstract

Predictions allow for efficient human communication. To be efficient, listeners’ predictions need to be adapted to the communicative context. Here we show that during speech processing this adaptation is a highly flexible and selective process that is able to fine-tune itself to individual language styles of specific interlocutors. In a newly developed paradigm, speakers differed in the probabilities by which they used particular sentence structures. Probe trials were applied to infer participants’ syntactic expectations for a given speaker and to track changes of these expectations over time. The results show that listeners fine-tune their linguistic expectations according to the individual language style of a speaker. Strikingly, nine months after the initial experiment these highly specific expectations could be rapidly reactivated when confronted with the particular language style of a speaker but not merely on the basis of an association with speaker identity per se. These findings highlight that communicative interaction fine-tunes and consolidates interlocutor specific communicative predictions which can overrule strong linguistic priors.

## Introduction

Human nature strongly relates to making predictions in various domains of cognitive processing^1–4^. In communication, more specifically the domain of language, predictions have been shown to affect the processing of phonological and visual word forms^5,6^ (but see^7^), semantic categories^8,9^, syntactic structure^10–12^ and event schemes^13^. Importantly, they are tightly coupled to incoming information, as the difference between a top-down prediction and the actual input updates representations at higher hierarchical levels^3,14^. By this mechanism, predictions remain adapted to the environment. For language, predictions are therefore shaped by language use itself^15,16^. Language users can hence be described as experts with highly adapted predictions about the form and structure of their native language. These predictions mirror the rules and statistical regularities of the respective language and can be thought of as linguistic priors^15^. Word order, for instance, constitutes such a regularity^17^. Even in languages which allow multiple word arrangements (e.g. German or Japanese), the preference to use one particular word order over the other creates a basic principle of language use. Listeners exploit this regularity by forming expectations about the word order of an upcoming sentence.

However, not every sentence is prototypical and language may vary widely depending on the speaker and the communicative situation. This contextual information needs to be taken into account in order to update internal representations and predictions. Context in language processing may be provided by purely linguistic information like sentence content^18^ or discourse context^19^, but also by extra-linguistic information like a speaker’s accent or language use^20–22^. For instance, Hanulíková *et al*. (2012) demonstrated that listeners’ ERP response to syntactic violations is decreased when violations are produced by a native speaker compared to a speaker with a foreign accent^22^. This shows that generalized knowledge inferred from a group of speakers can affect linguistic processing and is in line with the notion that listeners use multiple streams of information to understand language coming from linguistic and extralinguistic sources^23^. Exclusively relying on generalized knowledge, however, may not account for the variability one faces in everyday communication. On average we interact with eight different persons per day^24^. Each of these persons has an individual language style, which provides listeners with a rich “dataset” about preferred linguistic structure and content of a particular interlocutor^22,23^. Speaker identity is therefore a cue for communicative predictions which allow expectations about upcoming language input for a given speaker^4^.

We hypothesised that listeners adapt their expectations to the individual language use of a particular speaker and that this effect would manifest gradually with increasing exposure to the speaker. To test this hypothesis, we introduced a new experimental paradigm which allowed us to directly investigate syntactic expectations regarding sentence structure and to track changes of these expectations over time when coupled to a particular speaker. The paradigm uses the fact that language understanding adapts to the local statistics of the language environment, including the amount of exposure to particular syntactic structures in an experiment^15,25^. In the experiment two speakers produced German sentences which started either with a subject or an object. Following Kamide’s (2012) work on English^21^, the probability of a particular syntactic structure was manipulated depending on the speaker. In a within-subject design, one speaker had a high probability of producing subject-initial structures (*SOV Speaker*), while the other speaker had a high probability of producing object-initial structures (*OSV Speaker*).

Crucially, some of the presented sentences, the so-called probe trials, were ambiguous in their actual syntactic structure. In these probes the parts of the sentences necessary to parse the syntactic structure (i.e. the determiners) had been replaced with noise. Potential syntax related acoustic differences were counteracted by balancing the probes with regard to their original syntactic structure. By asking participants to identify either the subject noun or the object noun of these probe-sentences it was possible to infer the participant’s expectation regarding the syntactic structure of the sentence depending on the speaker. Note that there is a strong preference in German to use a subject-initial structure over an object-initial structure^26,27^. Thus, before encountering the language use of the particular speakers introduced in the experiment, participants were thought to expect the subject-initial structure on the basis of their linguistic priors.

**Figure 1:**
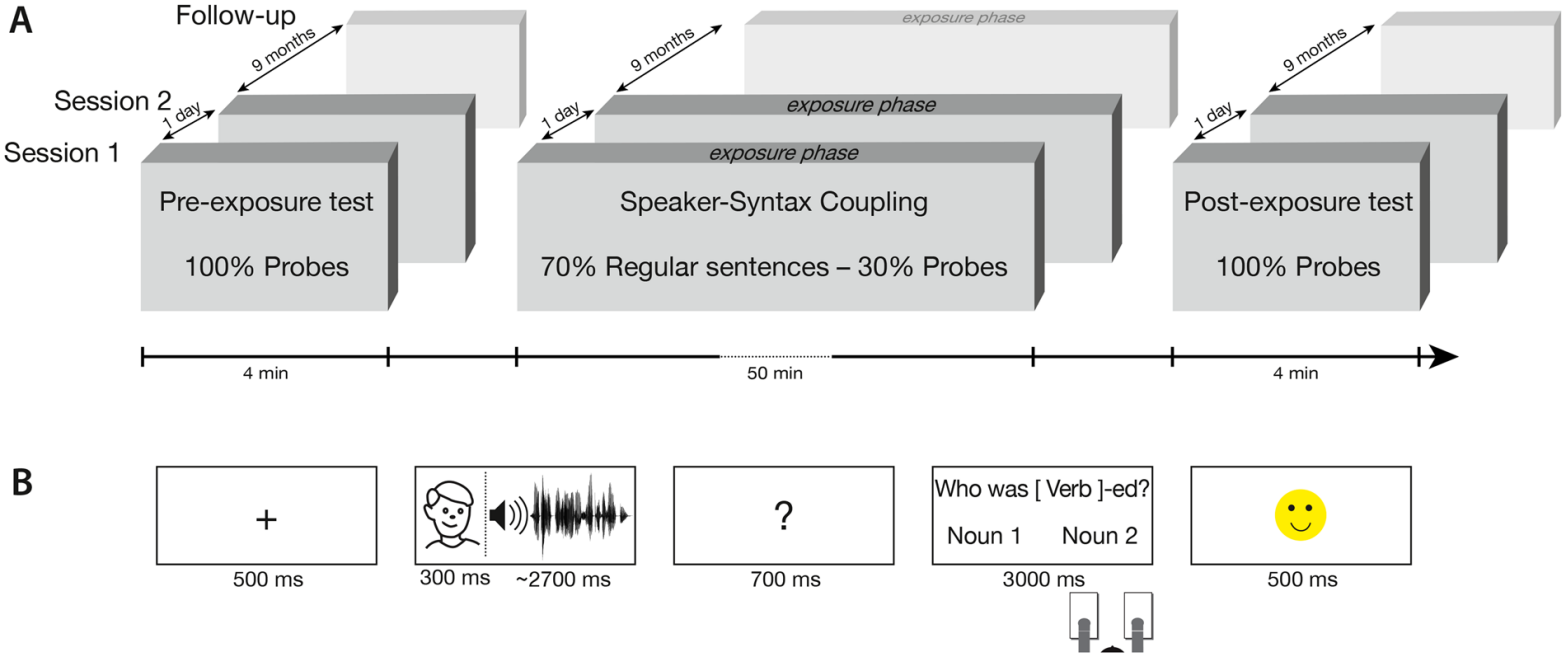
Experimental design and trial structure. Figure 1A gives a schematic overview of the experimental structure. All experimental sessions and the follow-up started with a pre-exposure test where only probe trials were presented. The crucial speaker-syntax coupling was established in the exposure phase, where regular and probe trials were pseudo-randomly interleaved. Every session was completed with a final post-exposure test where only probe trials were presented. Figure 1B shows a schematic trial structure for a regular trial.

Syntactic adaptation has been mostly studied in single session paradigms^15,21^. However, there is evidence that exposure to a particular syntactic structure can affect processing of this structure over the course of several days^28^, suggesting that adaptation results in long-lasting changes rather than in short-term priming. In the current study, we investigate whether speaker-specific adaptation relies on changes of expectations that persist beyond a single experimental session, by testing participants in two sessions on two consecutive days. Additionally, a post-hoc follow-up study was conducted nine months after the initial experiment to investigate long-term effects of the speaker manipulation. Every session consisted of an exposure phase which was framed by a pre-exposure test and a post-exposure test (Fig. 1A). In the critical exposure phase, regular sentences (i.e. sentences without noise) and probe sentences (i.e. sentences with noise) were pseudo-randomly interleaved. The regular sentence trials were used to establish the specific speaker-syntax coupling, while the probe trials were used to track participants’ expectations during the course of the exposure phase. The pre- and post-exposure test contained only probe trials and therefore allowed us to assess baseline levels in syntactic expectations before the exposure phase as well as the outcome of the speaker-syntax coupling on syntactic expectations after the exposure phase.

First, we analysed the pre- and post-exposure tests of Session one and two in order to assess the overall effect of exposure to a speaker’s individual language style on listeners’ syntactic expectations. We modelled participants’ response choices regarding the syntactic structure in the probe trials using mixed-effect logit models^29^. The results revealed a significant main effect of *Speaker* [χ^2^(1) = 9.528, p = 0.002], a significant main effect of *Test Position* [χ^2^(3) = 17.166, p < 0.001], and a significant interaction of *Speaker x Test Position* [χ^2^(3) = 16.367, p < 0.001]. The pre-exposure test of Session one was modelled as a baseline as it was the only position where participants had not encountered the speaker-syntax coupling before. Compared to this baseline, the Speaker effect (i.e. the difference between SOV Speaker and OSV Speaker) was increased in the post-exposure test of Session one ($\hat{\beta }$ = 0.542, Z = 3.025, p = 0.002), the pre-exposure test of Session two ($\hat{\beta }$ = 0.391, Z = 2.220, p = 0.026), and the post-exposure test of Session two ($\hat{\beta }$ = 0.694, Z = 3.880, p < 0.001). Post-hoc tests (Holm corrected) did not show a significant difference in the Speaker effect when comparing the post-exposure test of Session one to the pre-exposure test of Session two ($\hat{\beta }$ = −0.152, Z = −0.896, p = 0.37) or the pre-exposure test vs. the post-exposure test within Session two ($\hat{\beta }$ = 0.303, Z = 1.801, p = 0.143). This indicates that the mere exposure to the individual speaker styles modified participants’ expectations for the syntactic structure of sentences spoken by a particular speaker and that this adaptation remained in the second session on the consecutive day.

**Figure 2:**
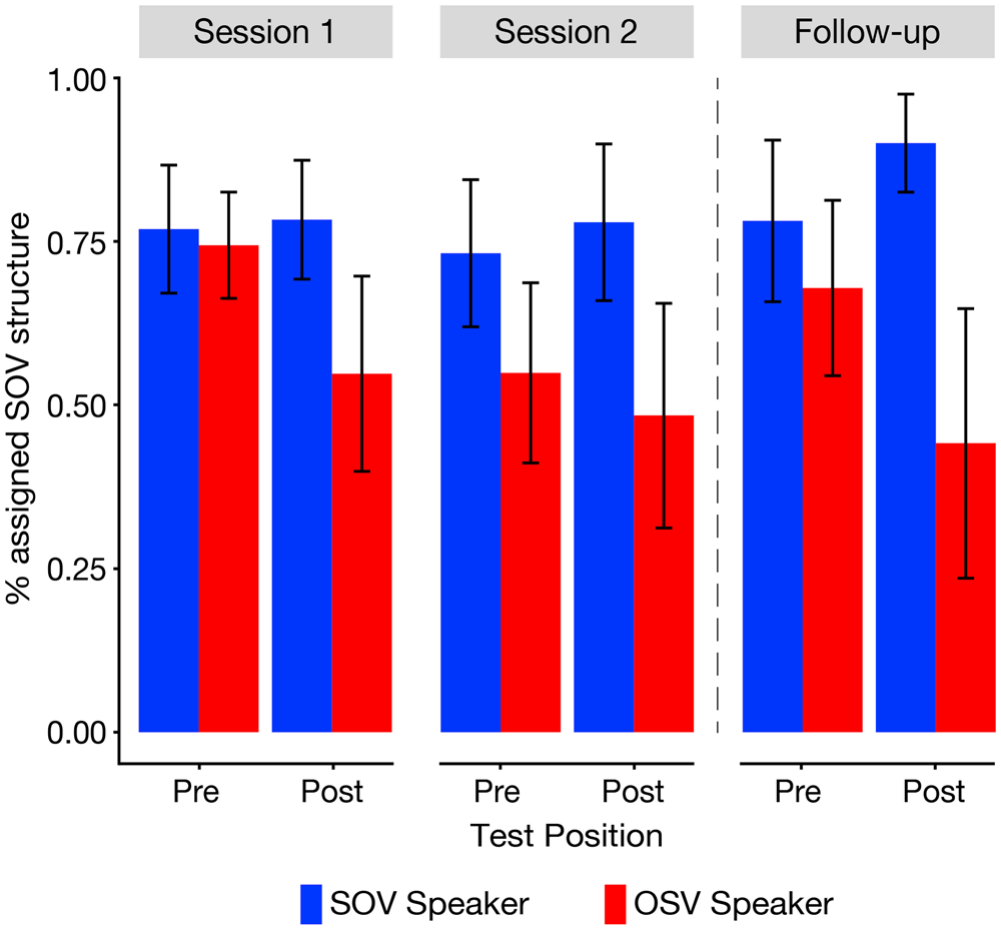
Percentage of assigned SOV structures in the pre- and post-exposure tests. Bars show the percentage of trials for which the SOV structure had been assigned to a probe sentence in the pre- and post-exposure tests of both sessions and both speakers and for the follow-up study. Error bars depict 95% confidence intervals.

The results of the follow-up study nine months later were similar to the first session (see Fig. 2). There was a significant main effect of *Speaker* [χ^2^(1) = 8.328, p = 0.003] and a significant interaction of *Speaker x Test Position* [χ^2^(1) = 32.299, p < 0.001]. A simple effect analysis revealed no difference between speakers at the pre-exposure test of the follow-up ($\hat{\beta }$ = 0.231, Z = 0.851, p = 0.395), but showed a significant difference between speakers at the post-exposure test of the follow-up $\hat{\beta }$( = 1.459, Z = 5.109, p < 0.001).

In order to integrate the results of the first two sessions and the follow-up we built an additional model containing all test positions of all sessions (cf. supplementary material). Post-hoc tests (Holm corrected) revealed (1) that the Speaker effect was significantly decreased at the pre-exposure test of the follow-up compared to the post-exposure test of Session two, nine months earlier ($\hat{\beta }$ = −0.409, Z = −2.293, p = 0.44) and (2) that the increase in the speaker effect from pre-exposure to post-exposure (i.e. the adaptation effect) was significantly greater in the follow-up compared to Session one ($\hat{\beta }$ = 0.541, Z = 2.002, p = 0.045). The results of the follow-up indicate that participants did not retrieve a syntactic structure with a particular speaker right away, but instead relied on the actual language use of that speaker in order to generate the expectations once more. Interestingly, speaker adaptation was more pronounced in the follow-up, suggesting that listeners could build on existing expectations.

**Figure 3:**
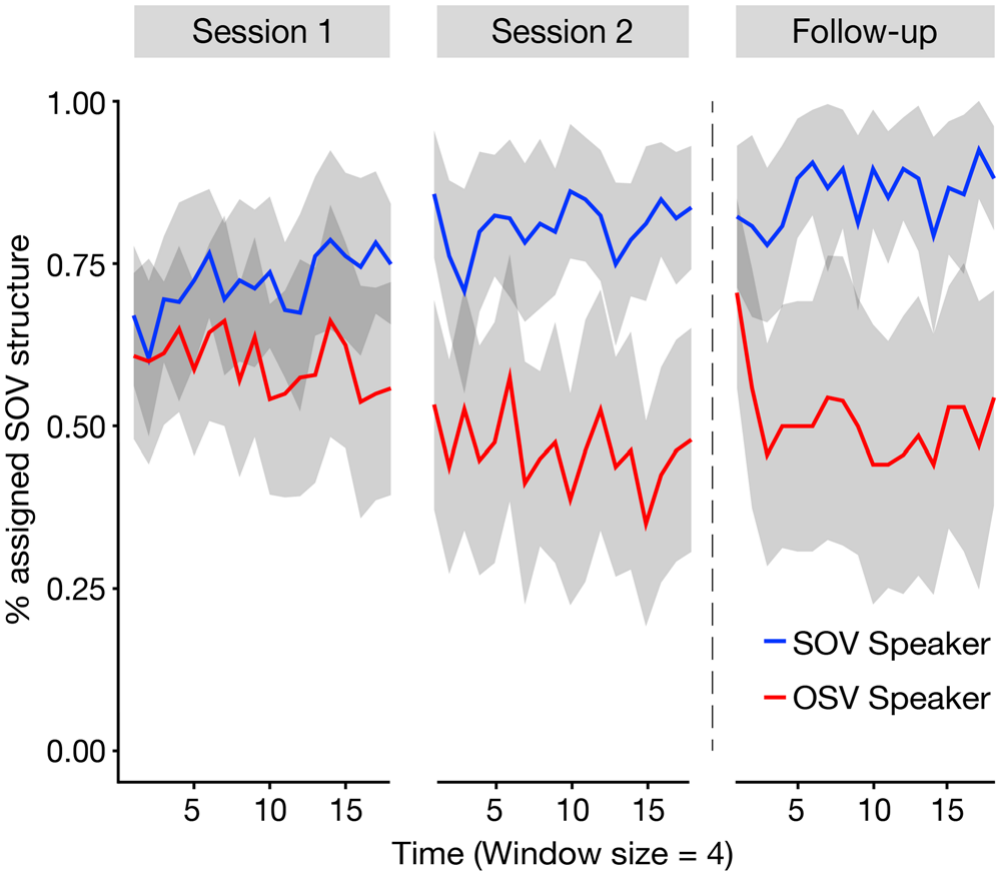
Percentage of assigned SOV structures in the exposure phase. The figure shows the percentage of trials for which the SOV structure had been assigned to a probe sentence during the exposure phase in both sessions and in the follow-up study. The data were subdivided into non-overlapping windows containing 4 trials per speaker (leading to 18 time points per session; see the supplement material for a description of the data without binning). For every window the proportion of ‘*SOV*’ responses was calculated. The shaded areas depict 95% confidence intervals.

In a next step we analysed the probe trials in the exposure phase since we were interested in the dynamic changes of the expectations over the course of the experimental sessions. Bear in mind that the probe trials were pseudo-randomly interleaved with regular sentence trials in the exposure phases of both sessions. As can be seen in Fig. 3, performance in these probe trials showed a steady adaptation to the speakers’ preferred syntactic structure, which was consolidated over sessions. The change in participants’ responses to the two speakers across the experiment was evaluated using growth curve analysis^30^. There was a significant main effect of *Speaker* [χ^2^(1) = 24.531, p < 0.001], a significant interaction of *Time* x *Speaker* [χ^2^(1) = 4.869, p = 0.027], and a significant interaction of *Speaker* x *Session* [χ^2^(1) = 7.416, p = 0.006]. The parameter of the *Time* x *Speaker* interaction revealed a linearly increasing difference in syntactic choices due to speaker over the course of the sessions [$\hat{\beta }$ = 0.015, t(77.73) = 2.207, p = 0.030]. Furthermore, the *Speaker* x *Session* interaction demonstrated an increased *Speaker* effect when comparing Session two to Session one [$\hat{\beta }$ = −0.268, t(60.46) = −2.663, p = 0.010]. These results show a gradual change in speaker-related expectations within and across experimental sessions, thereby demonstrating an online modification of syntactic expectations for particular speakers with increasing exposure to the speakers’ language style.

Again, the data of the follow-up study were analysed separately. The growth curve analysis revealed only a main effect of Speaker [χ^2^(1) = 15.835, p < 0.001], while the main effect of *Time* and the interaction of *Time x Speaker* were not significant. In order to describe the dynamics of the *Speaker* effect in more detail, we increased the temporal resolution by using windows of only two probe trials and then calculated separate growth curve analyses for the first five windows and the consecutive five windows using the same model specifications as above. The model containing the first five windows revealed a main effect of *Time* [χ^2^(1) = 8.104, p = 0.004], a main effect of *Speaker* [χ^2^(1) = 5.196, p = 0.023] and an interaction of *Time x Speaker* [χ^2^(1) = 4.543, p = 0.033], while the model containing the consecutive five windows revealed only a main effect of *Speaker* [χ^2^(1) = 12.682, p < 0.001]. The speaker effect did not emerge gradually as in Session one but was rapidly reinstated after a short period (within the first ten probe trials, cf. single trial data in supplementary material) of exposure to the speakers’ language style. This indicates that participants started to differentiate between speakers as soon as the speaker-syntax coupling was revealed in the regular trials.

Overall, our results demonstrate that listeners form speaker-specific syntactic expectations. These expectations were evident in probe sentences with an ambiguous sentence structure. Although these sentences were ambiguous, participants answered a subsequent comprehension question according to the most probable syntactic structure of the speaker producing the sentence. This response pattern persisted across days and was also present in a follow-up, nine months after the initial study. This finding rules out any short term priming of a syntactic structure as an explanation for our results^28^ and suggests a top-down use of statistical information present in the language input as a potential basis for speaker-specific syntactic expectations.

Before the experimental manipulation, participants did not differ in their syntactic expectancy between speakers since there was the usual preference to expect the subject-initial structure for both speakers in the baseline measurement. This is not surprising given the abundance of subject-initial structures in German^26^. In line with this, the results of the regular sentences showed evidence for increased processing difficulty for the OSV structure compared to the SOV structure (see also supplementary material). Such a “subject-first preference” has been previously discussed in a number of studies^31^. Electrophysiological studies, for instance, consistently report an increased late positivity (P600) when an ambiguous sentence is disambiguated in an object-initial structure compared to a subject-initial structure^31,32^, possibly reflecting processes of reanalysis or repair^33,34^. The P600 has also been discussed as the brain’s response to a failed prediction (i.e. prediction error)^35^. Taken together, these findings suggest that our native German participants had strong linguistic priors for a subject-initial structure.

Confirming our hypothesis, participants were able to modify these priors and generate expectations that were adapted to the syntactic preferences of a particular speaker. In the case of the SOV Speaker, the ratio between both structures was similar to the “real world”^27^ (90% SOV structures vs. 10% OSV structures). Therefore, the adaptation to this speaker was rather small. However, in the case of the OSV Speaker, the ratio between the structures was fundamentally different, in fact reversed, to what is found in everyday German language use^27^. Here, participants were able to adapt their expectations to this particular speaker after only one session of exposure to the speakers. Critically, we observed a steady tuning of participants’ expectations with increasing exposure to the two speakers (Fig. 3). These findings are in line with previous results about the adaptation of syntactic expectations^15,25,36–38^ and highlight the communicative role of speaker identity in the language comprehension system. By observing changes of speaker-specific expectations over multiple sessions, the study adds a long-term perspective to the existing literature on incremental syntactic adaptation^15,36,39^ and speaker adaptation^21,22^ (but see^40,41^). To our knowledge, the present study is the first to show speaker-specific syntactic expectations which are stable across different days.

Due to the offline nature of the current behavioural data, it remains unclear whether these syntactic expectations are processed in a forward or rather in a backward way. A forward way would suggest that expectations are integrated online during the build-up of a sentence representation, while a backward way would suggest that expectations are imposed on an existing sentence representation. Evidence from online reading experiments^15,36^, visual world eye-tracking experiments^21,25^ (but see^40,41^), as well as a recent ERP version of the current experiment with different participants^42^, however, suggest a forward mechanism of syntactic expectations.

The cue which allowed participants to generate syntactic expectations was speaker identity. As communication typically happens between persons, speaker identity is a salient source of information in language processing^20,43^. Previous studies have shown that social stereotypes or past experiences are generalised to individual speakers and affect language processing^20–23^. Our study adds to a growing body of work on incremental syntactic adaptation^15,36,39^ by showing additionally that listeners adapt their language processing in a fine-grained fashion to speaker-specific language use as soon as they encounter a new speaker. Furthermore, these adaptations can be demonstrated for something as fundamental as the expectancy for a specific syntactic structure^21,40,41^. Although speaker identity seems to be a very salient cue to inform linguistic expectations, we do not preclude that expectations may also be generated on the basis of other sources of information^44^. For example the proportion of valid semantic primes within an experimental block^45^ or the frequency of presentation of a syntactic structure^15,36^ could be helpful sources. It is therefore plausible to assume that expectations rely on multiple streams of information. However, as concerns communication, speaker identity might be in a prominent position and therefore be a salient and informative cue.

The fast and strong adaptation to a particular speaker in our initial experiment motivated a follow-up study to investigate whether speaker-specific predictions are still present nine months after initial exposure to the speakers. Interestingly, participants did not differentiate between speakers in the pre-exposure test of the follow-up where only probe stimuli were presented. However, as soon as the speaker-syntax coupling was reinstated, listeners immediately restored their syntactic expectations for a particular speaker and this adaptation effect was even more pronounced than in the initial exposure to the speakers in session one. It therefore seems that after nine months, speaker-specific expectations are not memorised ad hoc but can be re-established from existing representations when they become relevant in a communicative situation. Possibly this effect was driven by participants’ uncertainty in coupling an existing expectation to a particular speaker^20^. Thus, although the participants presumably remembered that there were two speakers in the experiment, they were uncertain which speaker had which syntactic preference. As soon as a minor amount of additional bottom-up information was given in the exposure phase, the listeners could explicitly re-establish the speaker-syntax coupling. An alternative explanation for the immediate restoration of the speaker-specific expectations would be that participants became faster in associating a speaker with a syntactic structure. In everyday life, however, language preferences are typically consistent within speakers thereby speaking against this hypothesis. Future studies might test this alternative by reversing the probabilities of a previously established speaker-syntax coupling. If the results can simply be explained by enhanced association build-up, the adaptation to the reversed coupling should be as fast as the adaptation to the original coupling.

Our results provide direct evidence that listeners adapt their syntactic expectations to the individual language style of a particular speaker. This adaptation is an online process that starts as soon as a listener is confronted with utterances of an interlocutor. With increasing evidence for a particular language style, a listener’s expectations are then fine-tuned to meet the characteristics of a speaker’s language preference, thereby possibly giving communicative benefits. Furthermore, these expectations can be reinstated almost immediately nine months later. That speaker-specific syntactic expectations persist across days and months suggests that the brain might actually maintain statistical information related to language input and possibly keep doing so for particular speakers throughout our adult life. Our results therefore highlight the role of speaker-specific top-down predictions in the language comprehension system.

## Methods

### Participants

Twenty healthy volunteers participated in this study (mean age = 24.35 years, age range 21–29 years, 11 women). One participant was excluded from the analysis of the pre- and post-exposure test due to technical problems. All participants were healthy, native German speakers with normal or corrected-to-normal vision and did not report any hearing deficits. Participants gave written informed consent and received 14 € compensation. Experimental procedures were approved by the ethics committee of the University of Leipzig (159/16-ek). The study was conducted according to the approved guidelines.

## Materials

Experimental items consisted of short German sentences, which had either a subject-initial *Subject-Object-Verb* (SOV) structure or an object-initial *Object-Subject-Verb* (OSV) structure (Table 1). The sentence structure was specified by the case-marking of the determiners. A nominative determiner (i.e. *der*) indicated a subject noun phrase, while an accusative determiner (i.e. *den*) indicated an object noun phrase. The syntactic structure of a sentence became clear at the determiner of the first noun phrase. Every noun was implemented both as the subject and as the object in different versions of a particular sentence item. This was possible because all nouns were selected to be semantically plausible both as subject and as object of a sentence. Sentences were generated from a set of 240 items. Every item was realised with both sentence structures (SOV vs. OSV) and with both nouns being implemented as subject or object. This led to 960 experimental sentences.

**Table 1:** Sentence material. Four experimental sentences were constructed per item.

**Structure**	**Version 1**	**Version 2**
**SOV**	Heute hat der Mann den Freund gesehen.	Heute hat der Freund den Mann gesehen.
	*Today has the* [nom.] *man the* [acc.] *friend seen*.	*Today has the* [nom.] *friend the* [acc.] *man seen*.
**OSV**	Heute hat den Freund der Mann gesehen.	Heute hat den Mann der Freund gesehen.
	*Today has the* [acc.] *friend the* [nom.] *man seen*.	*Today has the* [acc.] *man the* [nom.] *friend seen*.
**Probe**	Heute hat XXX Mann XXX Freund gesehen.	Heute hat XXX Freund XXX Mann gesehen.
	*Today has XXX man XXX friend seen*.	*Today has XXX friend XXX man seen*.

These sentences differed in their syntactic structure (SOV vs. OSV) and in whether a noun was the subject or the object of the sentence. The syntactic structure of a sentence became clear at the determiner of the first noun phrase (i.e. der or den). For the probe trials the determiners were replaced with white noise (XXX indicating the noise). For every item the same nouns were implemented both as subject and object of the sentence (Version 1 and 2).

All sentences were recorded by a professional female and male speaker (sampling rate 44.1 kHz, Audacity 2.0). Speakers were instructed to utter the sentences with normal speed and without emphasising one noun over the other. After the recordings, sound files were processed using Adobe Audition 3.0. A 50 ms silence period was inserted at the beginning and the end of every sentence and at the onset of the first determiner. Sentence amplitude was normalised using the root mean square. This led to a total of 1,920 stimuli with an average length of 2,717 ms (SD = 221 ms). These stimuli will be referred to as regular sentences, as they conveyed information both about the speaker and the syntactic structure. Additionally, a probe version was created for every sentence by replacing the determiners with white noise. With the case-marking of the determiners missing, the resulting sentences were ambiguous with regard to their syntactic structure. Importantly, these stimuli still conveyed information about the speaker but did not provide any information about the syntactic structure of the sentences.

In the experiment, auditory sentences were always presented together with a picture of a female or a male face (depending on the speaker’s gender). These two stimuli were taken from the NimStim set of facial expressions (neutral and “mouth closed” conditions, MacBrain Face Stimulus Set^46^).

### Procedure

On two consecutive days, participants performed two sessions which had the same experimental procedure. A randomised list was created for every participant and session and items were assigned randomly into conditions. Sessions consisted of a pre- and a post-exposure test, where only probe sentence trials were presented, as well as an exposure phase, where both regular and probe sentence trials were presented. The purpose of the pre- and post-tests was to measure participants’ predictions before and after exposure to the speaker-syntax coupling. The purpose of the exposure phase was to establish predictions in the form of a speaker-syntax coupling and to track these predictions over the course of an experimental session using probes. Participants received written instructions concerning the experimental procedure. They were told to listen to the sentences and answer comprehension questions which asked randomly either for the subject or the object of the previously presented sentence. With regard to the probes, they were told to answer the questions according to what they thought the original version (without noise) of the sentence was.

Twenty probe trials were presented in the pre-exposure test and another twenty were presented in the post-exposure test (ten per speaker). The exposure phase consisted of 480 trials. Of these trials 336 were regular sentence trials (70%) and 144 were probes (30%) which were evenly distributed across the exposure phase. Over all trials the total number of SOV and OSV structures and the total number of sentences spoken by the male and female speaker was balanced. Although there was no overtly recognisable syntactic structure in the probe sentences, we balanced sentences within speakers with regard to the syntactic structure of the original regular sentence from which they had been generated in order to control for potential acoustic cues that might have indexed a particular structure.

For the regular sentences, the speakers differed with regard to the frequency of sentences with SOV and OSV structures. In the case of the so-called “SOV Speaker”, 152 sentences were presented with a SOV structure (90.48%) and only 16 sentences were presented with an OSV structure (9.52%). However, in the case of the “OSV Speaker” 152 sentences were presented with an OSV structure (90.48%) and only 16 sentences were presented with a SOV structure (9.52%). The association between speaker gender and syntactic structure was balanced across participants. This means that for half the participants the male speaker was the OSV Speaker and the female speaker was the SOV Speaker and vice versa for the other half of the participants.

Trial structures were similar for regular and probe trials (Fig. 1B). Every trial started with the presentation of a fixation cross for 500 ms. Next, the face of the speaker was presented on the screen. After 300 ms the sentence was presented via headphones while the face of the speaker remained on the screen for the duration of the sentence. Following sentence presentation, a question mark appeared on the screen for 700 ms. After this a comprehension question was presented. The question either asked for the object of the sentence (“*Who was* [verb]-*ed?*”/“*Wer wurde ge*-[verb]*-t*?”), or for the subject of the sentence (“*Who did* [verb]?”/“*Wer hat ge-*[verb]*-t?*”). The two nouns of the previous sentence were presented below the question on the left and the right side of the screen, respectively. The question type as well as the side of the correct answer was balanced over trials. Participants had to respond within 3,000 ms. In the regular sentence trials feedback was presented after the response for 500 ms. In the probe trials there was no feedback.

### Task

Although the same comprehension questions were used for regular and probe trials, they had very different functions in the experiment. For the regular trials, responses could be either correct or incorrect. This allowed us to ensure that participants were focusing on the stimuli and to measure performance.

For the probe trials, however, the responses were neither correct nor incorrect as the probe sentences did not specify the subject and the object of the sentence. Still, participants had to answer a comprehension question which asked either for the subject or the object of the previous sentence. The selected noun marked the noun which had been interpreted as the subject or as the object of the sentence (depending on the question type). This information in combination with the position of that noun in the previous sentence allowed us to infer whether the probe sentence had been parsed as a SOV structure or as an OSV structure. For example, if a participant had selected the noun “*man”* as the subject of a probe sentence like “*Today has [XXX] man [XXX] friend seen*.”, the sentence must have been parsed as a SOV structure because “*man”* is the first noun in the sentence. Conversely, if a participant had selected the noun “*friend”* as the subject of the same sentence, the sentence must have been parsed as an OSV structure because “*friend”* is the second noun in the sentence. The percentage of trials which were parsed as a SOV structure was taken as dependent variable. This allowed us to infer participants’ syntactic expectations with regard to the speaker of the sentence, even when no syntactic information was available in the stimuli.

### Follow-up

A follow-up study was conducted nine months (mean = 9.2 months, SD = 0.2 months) after the initial experiment in order to assess the long-term significance of the observed speaker effects. Seventeen participants of the initial cohort were recruited to participate in the follow-up. The follow-up study consisted of only one session and the stimulus presentation order was again randomised. Apart from that, stimuli and experimental procedure of the follow-up study were identical to the first session of the initial experiment. This study was planned post-hoc to the initial experiment and was therefore not included in the factorial design, but instead analysed separately.

### Statistical analysis

All statistical analyses were performed in the R environment^47^ with packages *lme4* ^48^, *car* ^49^, and *lmerTest* ^50^ installed.

The analysis of the responses in the pre- and post-exposure tests was conducted using mixed-effects logit models^29^ in order to accommodate the categorical nature of the data. The model of the initial experiment (containing the first two sessions) included fixed effects for *Speaker* (2 levels: SOV Speaker, OSV Speaker), *Test Position* (4 levels: Pre-and post-exposure of both sessions), and the interaction of *Speaker x Test Position*. The maximal random effects structure for which convergence was reached^51,52^ included random intercepts by subjects, random intercepts by Item as well as random slopes for *Speaker* by subjects. The factor *Test Position* was simple coded with the pre-exposure test of the first session as a baseline (four levels of *Test Position* thus resulted in three contrasts). Note that the pre-exposure test of the first session was a real baseline, as this was the only test conducted before the participants had been exposed to the speaker-syntax coupling. The factor *Speaker* was sum coded (SOV-Speaker: 1 vs. OSV-Speaker: −1). The resulting parameter estimates of the interaction of *Speaker x Test Position* thus test whether the difference between speakers is different at a particular test position compared to the difference between speakers at baseline. For the follow-up study the same model was defined, but *Test Position* only had two levels (Pre- and post-exposure). Both factors were sum coded (SOV-Speaker: 1 vs. OSV-Speaker: −1 and pre-exposure test: 1 vs. Post-exposure test: −1). For all models possible main effects and interactions were evaluated using Type II Wald chi-square tests and parameter estimates were evaluated using Wald tests. A simulation approach^53^ with an odds ratio of 1.68 as effect size (equivalent to Cohen’s d = 0.2^54^), an α-error of 5%, and n = 19 revealed a power of 1-β = 91.8% (95% CI: 89.92–93.43) when assessing the *Speaker* effect.

The analysis of the responses in probe trials within the exposure phase was conducted using growth curve analysis^30^. To evaluate changes over the course of the experiment, participants’ response behaviour was modelled using a linear term. Responses in the probe trials were subdivided into non-overlapping, consecutive windows of four trials per speaker (18 time points per speaker per session) and the proportion of ‘*SOV*’ responses within windows was taken as dependent measure. As the underlying data were still categorical, the empirical logit transformation was used^55^. These data were entered into a linear mixed-effect model with fixed effects for *Speaker* (2 levels: SOV Speaker, OSV Speaker), *Session* (2 levels: Session one, Session two), and *Time* (continuous regressor: a time point relates to a single window composed of four trials) and the interactions between all factors. The model also included two sets of random effects, one at the subject level (with random intercepts by subject and random slopes for *Time* by subject) and one at the lowest level of nesting in the data (with random intercepts by the interaction of Subject x *Speaker* x *Session* and random slopes for *Time* by the interaction of Subject x *Speaker* x *Session*)^30^. The factors *Speaker* and *Session* were sum coded (SOV-Speaker: 1 vs. OSV-Speaker: −1 and Session one: 1 vs. Session two: −1). For the follow-up study the same growth curve analysis was conducted, but without the factor *Session* for obvious reasons. For both models, possible main effects and interactions were evaluated using Type II Wald chi-square tests. Parameter estimates of the linear mixed-effect models were evaluated using t-tests with Satterthwaite approximations to the degrees of freedom^56^.

### Data policy

Data in an anonymized form (in accordance to the ethics agreement) and scripts used in data analysis are available on request.

## Electronic supplementary material

Supplementary Material
