# Supplementary material for: Communicative predictions can overrule linguistic priors
Source: Sci Rep. 2017 Dec 14;7:17581. doi: 10.1038/s41598-017-17907-9 (PMC5730607; doi:10.1038/s41598-017-17907-9)
Supplement: Supplementary file 1 — Supplementary Material [file 41598_2017_17907_MOESM1_ESM.pdf]

Communicative predictions can overrule linguistic priors

Supplementary Material

Leon O.H. Kroczek*[1] & Thomas C. Gunter[1]

[1]Max Planck Institute for Human Cognitive and Brain Sciences, Leipzig, Germany

Performance in the comprehension task of regular trials

Participants performance on the comprehension task was analysed in order to investigate the effects of syntactic structure (Figure S1). Response types (correct vs. incorrect) were modelled using a mixed-effect logit model with fixed effects included for the factors *Structure* (SOV vs OSV) and *Session (*Session one vs. Session two*)* and a full random effects structure. All factors were sum coded. The model revealed a main effect of *Structure* [$\chi^2(1) = 27.911$, $p < .001$] with an increased error rate for OSV compared to SOV sentences, and a main effect of *Session* [$\chi^2(1) = 49.246$, $p < .001$] with a decreased error rate in Session two compared to Session one. There was no interaction between both factors [$\chi^2(1) = 0.273$, $p = .601$]. Reaction times were modelled using a linear mixed-effect model with the same fixed and random effect structure as the model used for the response types. The model revealed a significant main effect of *Session* [$\chi^2(1) = 24.681$, $p < .001$] , but no main effect of *Structure* [$\chi^2(1) = 0.876$, $p = .35$] and no interaction [$\chi^2(1) = 1.192$, $p = .275$.].

Data were analysed separately for the follow-up study using the same model specifications as above (only without the factor *Session*). The results of the response types (correct vs. incorrect) show a significant main effect of *Structure* [$\chi^2(1) = 28.999$, $p < .001$] with increased errors for OSV sentences compared to SOV sentences. With regards to reaction times there was no effect of *Structure* [$\chi^2(1) = 2.571$, $p = .101$].

Overall, performance in the comprehension task of regular sentences (i.e. non-probe trials with all syntactic information available) improved over sessions and showed the typical processing advantage for easy subject-initial sentences compared to complex object-initial sentences. This demonstrates that listeners were attentive to the stimuli and engaged in normal sentence processing.

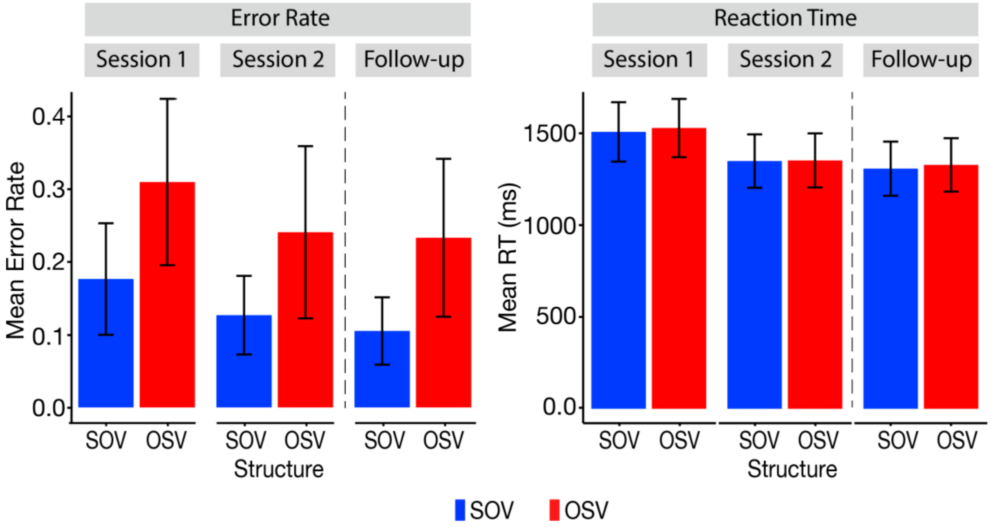

Figure S1: Results for error rates (left) and reaction times (right) in the comprehension task for regular sentences. Averages are shown for both syntactic structures in both sessions and the follow-up study. Error bars reflect the 95% confidence Intervals.

Model including pre- and post-exposure tests of session one/two and the follow-up

In order to integrate findings from the first two sessions and from the the follow-up nine months later an additional model was calculated. The responses in the pre- and post-exposure tests were modelled using a logit mixed effect model with fixed effects for the factor *Speaker* (SOV Speaker vs. OSV Speaker) and *Test Position* (six level: pre- and post-exposure tests from all sessions). The model also included random intercepts by subject, random intercepts by *Item* as well as random slopes for *Speaker* by subjects. The factor *Test Position* was simple coded with the pre-exposure test of the first session as a baseline The factor *Speaker* was sum coded (SOV-Speaker: 1 vs. OSV-Speaker: -1). Therefore, all interaction parameters compared the speaker effect at consecutive test positions to the speaker effect at pre-exposure baseline. The model revealed a main effect of *Structure* [$\chi^2(1)$ = 11.185, p < .001], a main effect of *Test Position* [$\chi^2(5)$ = 20.393, p = .001], and an interaction of *Speaker x Test Position* [$\chi^2(5)$ = 51.498, p < .001]. The parameter estimates revealed that overall participants were more likely to assign a SOV structure to a sentence, when the sentence was produced by the SOV Speaker compared to the OSV Speaker ($\hat{\beta}$ = 0.570, Z = 3.624, p < .001). Compared to the baseline, the *Speaker* effect was significantly increased for all test positions but the pre-exposure test of the follow-up (Post-Session one: $\hat{\beta}$ = 0.555, Z = 3.119, p = .001, Pre-Session two: $\hat{\beta}$ = 0.379, Z = 2.200, p = .028, Post-Session two: $\hat{\beta}$ = 0.672, Z = 3.845, p < .001, Pre-Follow-up: $\hat{\beta}$ = 0.263, Z = 1.414, p = .157, Post-Follow-Up: $\hat{\beta}$ = 1.358, Z = 6.654, p < .001). In addition, a simple effects analysis revealed significant differences between speakers at all test positions but the pre-exposure test of session one and the pre-exposure test of the follow-up (Pre-Session one: $\hat{\beta}$ = 0.032, Z = 0.163, p = .87, Post-Session one: $\hat{\beta}$ = 0.587, Z = 3.045, p = .002, Pre-Session two: $\hat{\beta}$ = 0.411, Z = 2.198, p = .028, Post-Session two: $\hat{\beta}$ = 0.704, Z = 3.721, p < .001, Pre-Follow-up: $\hat{\beta}$ = 0.295, Z = 1480, p = .139, Post-Follow-up: $\hat{\beta}$ = 1.390, Z = 6.439, p < .001).

Taken together, these results demonstrate that adaptation towards the speaker-specific language use emerged in Session one and was maintained over Session two. Interestingly, this speaker-specific adaptation was not present at the the pre-exposure test of the follow-up, but was re-instated after the exposure phase.

Responses in single probe trials over the course of the experiment

Figure S2 shows the underlying raw data used in the growth curve analysis. Every data point reflects the percentage of participants who responded with a *SOV* response for a given single probe trial. The observed pattern corresponds to the pattern reported for the growth curve analysis using windows of four probe trials.

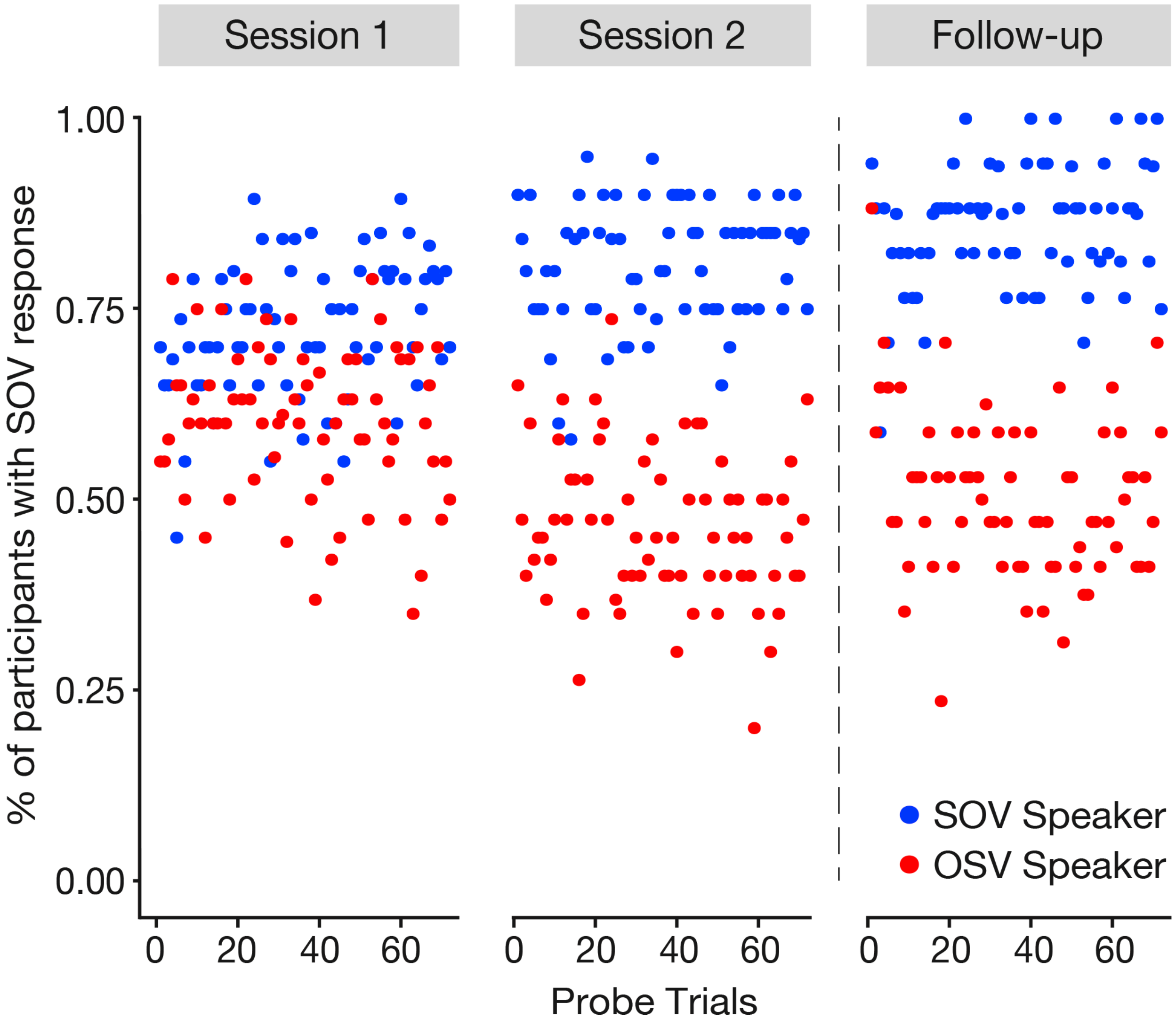

Figure S2: The percentage of participants with a *SOV* response in single probe trials across the exposure phases. Probe trials are ordered according to their position within the session. Please note, that these are the raw data underlying the four trial windows used in the growth curve analysis.
